# Multi-omics analysis reveals the specific role of biocontrol reagents against tomato bacterial wilt

**DOI:** 10.3389/fpls.2025.1620460

**Published:** 2025-07-14

**Authors:** Xin-Qiao Du, Tian-Xiao Sun, Wu-Lin Xu, Tang Zhu, Qiang Wang, Pei-Wen Gu, Jiang Lu

**Affiliations:** ^1^ School of Agriculture and Biology, Shanghai Jiao Tong University, Shanghai, China; ^2^ Chengdu Topu Biotechnology Co., LTD, Chengdu, China; ^3^ Chengdu Juzhuo Biotechnology Co., LTD, Chengdu, China; ^4^ Sichuan Taikang Biotechnology Co., LTD, Chengdu, Yinchuan, China; ^5^ School of Agriculture, Ningxia University, Yinchuan, China

**Keywords:** bacterial wilt (*Ralstonia solanacearum*), biocontrol agent, transcriptomics, metabolomics, bacterial community

## Abstract

Bacterial wilt caused by *Ralstonia solanacearum* is considered one of the most important diseases that cause economic losses to tomato. Currently, eco-friendly biocontrol agents have been increasingly considered as effective approaches to control tomato bacterial wilt. However, the specific mechanisms by which biocontrol bacteria with distinct functions exert their effects remain unclear. In this study, we employed a combination of amplicon sequencing, transcriptomics, and metabolomics analysis to investigate how *Bacillus velezensis* and *Pseudomonas fluorescens* affect the defense responses against *R. solanacearum* in tomato. We showed that the fermentation broth of these biocontrol agents inhibited the growth of *R. solanacearum in vitro*, and improves the ability of tomato plants against bacterial wilt. In general, different biocontrol agents protect plants from bacterial wilt in many ways, by recruiting specific microbial communities in rhizosphere soil and activating different synthetic/metabolic and signaling pathways. Collectively, our findings contribute to a more in-depth understanding in disease resistance mechanisms of biocontrol agents, and provide a theoretical foundation for the development of targeted strategies using beneficial microorganisms to suppress disease occurrence.

## Introduction

Tomato (*Solanum lycopersicum* L.), the second-most consumed vegetable crop in the world, encounters many soil microbial infestations which reduce the quality of plants and lead to large losses in production (Nicola et al., 2009). Bacterial wilt caused by *Ralstonia solanacearum* (*Rs*) is a severely damaging vascular disease that spreads worldwide and infects a wide host range including tomato (Wei et al., 2024a). *R. solanacearum* enters mainly through root wounds, establishes colonies, reaches the xylem through the intercellular space, and eventually fills the entire xylem duct, resulting in necrosis and wilting of the infected plants (Shi et al., 2022).

For many decades, chemical pesticides are commonly used to control crop disease and ensure agricultural harvest. However, the long-term continuous spraying of pesticides will cause pesticide residues of agricultural product, environmental pollution and risk on public health (Wang et al., 2023). To solve this problem, environmentally friendly controls are recommended in order to avoid the adverse effects of chemical pesticides (Raza et al., 2016; Soesanto et al., 2019). Among them, biological control by the application of natural antagonists of pathogenic bacteria can be an option for disease management. Many beneficial microbes such as *Pseudomonas fluorescens*, *Bacillus velezensis*, and *Bacillus amyloliquefaciens*, have been documented as effective biocontrol agents against *R. solanacearum* (Sotoyama et al., 2017; Soesanto et al., 2019; Sharma et al., 2022; Dong et al., 2023).

These beneficial microbes endow to plants with antibacterial peptides (e.g., bacteriocins) and antibacterial metabolites (e.g., antibiotics) that protect plant from pathogenic attacks. Similarly, they also trigger a wealth of molecular processes in plants, including the production of osmolytes like soluble sugars, to increase the plant tolerance to biotic stresses (Bastías et al., 2022). Furthermore, many beneficial microbes stimulate host immune responses by priming jasmonic acid (JA)/ethylene (ET) and salicylic acid (SA) signaling pathways in plants, thereby playing an important role in controlling bacterial wilt disease (Yang et al., 2023). For instance, the expression of plant defense marker genes such as *PR1*, *PR2*, *PR3*, *PR5*, *PR12*, *ERF1*, and *LoxA* in the SA and JA/ET signaling pathways, as well as genes like *FLS2* and *MKK9* in the MAPK signaling pathway, was significantly up-regulated in infected tomato plants treated with beneficial microbes (Le et al., 2021; Kim et al., 2022; Vargas et al., 2022; Wei et al., 2024b).

High-throughput sequencing techniques including genomics, metabolomics, transcriptomics, and proteomics were widespread used to unravel the intricacies involved in plant-pathogen interactions (Ramlal et al., 2023). Among them, metabolome and transcriptome analysis provide a rapid and efficient way to identify key metabolites and regulatory pathways in plants responding to bacterial wilt (Shi et al., 2022). Using these approaches, glutathione metabolism and phenylpropane pathways of tobacco were demonstrated as primary resistance pathways to *R. solanacearum* infection, and resistance-related genes responds to *R. solanacearum* were identified (Li et al., 2021; Yang et al., 2022). However, how different biocontrol agents regulate plant-*R. solanacearum* interaction and their specificity need to be further elucidated, which is crucial for developing effective control strategies.

In this study, we tested the antagonistic activity of biocontrol agents, including *B. velezensis* and *P. fluorescens*, against *R. solanacearum in vitro* and in the field. Multi-omics analysis including microbiome, transcriptome and metabolome was used to elucidated the mechanism underlying the ability of these biocontrol agents to promote the growth of tomato plants and confer resistance to bacterial wilt. The results showed that different biocontrol agents may recruit specific microbial communities and activate specific biological processes to improve the disease resistance of tomato plants. The identification of the pathways and compounds associated with defense responses will not only increase our understanding of the tomato-*R. solanacearum* interaction affected by biocontrol agents, but also facilitate the improvement of resistance breeding.

## Materials and methods

### Determining *R. solanacearum* pathogen inhibition by biocontrol bacteria

In order to study the negative effects of biocontrol bacterial on the pathogen, we compared the reduction in pathogen density when cultured in the fermentation broth of biocontrol bacteria versus when grown alone. We used *R. solanacearum* strain Rs01 (kindly provided by Prof. Xin Zhang, Institute of Plant Protection, Jiangsu Academy of Agricultural Sciences) as the tomato pathogenic bacterium, and biocontrol bacteria including *B. velezensis* strain LJBv226 and *P. fluorescens* strain LJPf01 in our experiments. These bacteria were routinely grown at 30°C in NB medium (glucose 10.0 g/L, peptone 5.0 g/L, yeast extract 0.5 g/L, beef extract 3.0 g/L) with shaking (200 rpm) before all the experiments.

The biocontrol bacteria were cultured in NB medium for 24 h as described above with the addition of centrifugation (10 min at 8000 rpm) and filtration (0.45 μm) steps to prepare the fermentation broth. The *R. solanacearum* (200 μL, 3.8×10^8^ CFU/mL, OD_600_ = 2) was inoculated into these two kinds of fermentation broth (10 mL). After 0 h and 24 h of cultivation, pathogen densities were measured as optical density (OD_600_ (nm)) using a spectrophotometer. The *R. solanacearum* (200 μL, 3.8×10^8^ CFU/mL, OD_600_ = 2) was also grown alone in NB medium (10 mL) as the control and all treatments were measured with 12 biological replicates.

### Evaluation of the ability of biocontrol agents antagonistic tomato bacterial wilt in the field

The field experiment was performed from August to October 2024 at a tomato field in Huaiyin District of Jiangsu Province, China, where tomato bacterial wilt breaks out every year. The granules, produced by Chengdu Tepu Biotechnology Co., Ltd., contains 2×10^8^ CFU/g *B. velezensis* or 5×10^8^ CFU/g *P. fluorescens*. The tomato seedlings were transplanted into the field on August, 4. Before the onset of tomato bacterial wilt, The biocontrol agents were diluted to 1:150, 1:300 and 1:500, respectively, and sprayed to the roots (50 mL per tomato plant) for the first time (September, 12). and then twice again at the flowering stage (September, 15 and 18). Finally, the disease incidents, biocontrol efficiency, plant height, root length, stem diameter, and chlorophyll content (SPAD) were investigated at 15 d after the application of biocontrol agents (September, 27). Tomato plants without biocontrol agents were used as control. Disease incidence and biocontrol efficiency were calculated by formula as follows:

Disease incidence (%) = (Total number of infected plants/Total number of plants) ×100;
Biocontrol efficiency (%) = (Disease incidence of control - Disease incidence of treatment)/Disease incidence of control ×100

### Rhizosphere sample collection and DNA extraction

Rhizosphere soil samples were collected 15 days after the application of biocontrol regents, according to the method described by Wei et al. with some modifications (Wei et al., 2024a). Specifically, tomato plants were carefully uprooted and the loose soil was shaken off, and then the soil attached to the root surface (within approximately 0–4 mm) was collected as rhizosphere soil. After removing the debris and residual fine roots, the soil samples collected in the field were sealed in sterile self-sealed bags, immediately placed in an ice box, and stored in a -80°C freezer until the determination of soil community structure.

Soil (0.25 g) from each sample was used for DNA extraction using the DNeasy PowerSoil Kit (QIAGEN, Inc., Netherlands), following the manufacturer’s instructions. The extracted DNA was checked on 1.2% (w/v) agarose gels, and quantified using a NanoDrop ND-1000 spectrophotometer (Thermo Fisher Scientific, Waltham, MA, USA).

### Illumina Novaseq high-throughput sequencing and statistical analysis for microbial communities

PCR was performed to amplify the bacterial 16S rRNA genes V3–V4 region using the forward primer 338F (5’-ACTCCTACGGGAGGCAGCA-3’) and the reverse primer 806R (5’- GGACTACHVGGGTWTCTAAT-3’) (Claesson et al., 2009) or amplify the fungal internal transcribed spacer (ITS) sequences using the forward primer ITS1 (5’-GGAAGTAAAAGTCGTAACAAGG-3’) and the reverse primer ITS2 (5’-GCTGCGTTCTTCATCGATGC-3’) (Li et al., 2016). Sample-specific 7-bp barcodes were incorporated into the primers for multiplex sequencing. The PCR components contained 5 μL of Q5 reaction buffer (5×), 5 μL of Q5 High-Fidelity GC buffer (5×), 0.25 μL of Q5 High-Fidelity DNA Polymerase (5U/μL), 2 μL (2.5 mM) of dNTPs, 1 μL (10 μM) of each Forward and Reverse primer, 2 μL of DNA Template, and 8.75 μL of ddH_2_O. Thermal cycling consisted of initial denaturation at 98°C for 2 min, followed by 25 cycles consisting of denaturation at 98°C for 15 s, annealing at 55 °C for 30 s, and extension at 72°C for 30 s, with a final extension of 5 min at 72°C. PCR products were measured through agarose gel electrophoresis, and purified with Vazyme VAHTSTM DNA Clean Beads. Library construction was performed according to the standard procedure of TruSeq Nano DNA LT Library Prep Kit for Illumina^®^. The constructed library was sequenced on an Illumina Nova 6000 platform at Irun Biological Technology Co., Ltd. (Shanxi, China), using Miseq.

Adaptors and primer sequences were removed using Cutadapt and FASTP (version 0.14.1) software to obtain clean reads. The raw sequences were then denoised by the use of DADA2. The Quantitative Insights Into Microbial Ecology (QIIME, version 2) pipeline was employed to process the sequencing data, as previously described (Caporaso et al., 2010). Briefly, raw sequencing reads with exact matches to the barcodes were assigned to respective samples and identified as valid sequences. The low-quality sequences were filtered through following criteria (Chen and Jiang, 2014): sequences that had a length of <150 bp, sequences that had average Phred scores of <20, sequences that contained ambiguous bases, and sequences that contained mononucleotide repeats of >8 bp. Paired-end reads were assembled using FLASH (Magoč and Salzberg, 2011). After chimera detection, the remaining high-quality sequences were clustered into operational taxonomic units (OTUs) at 97% sequence identity by UCLUST (Edgar, 2010). A representative sequence was selected from each OTU using default parameters. OTU taxonomic classification was conducted by BLAST searching the representative sequences set against the Greengenes Database (DeSantis et al., 2006) using the best hit (Altschul et al., 1997). An OTU table was further generated to record the abundance of each OTU in each sample and the taxonomy of these OTUs. OTUs containing less than 0.001% of total sequences across all samples were discarded. To minimize the difference of sequencing depth across samples, an averaged, rounded rarefied OTU table was generated by averaging 100 evenly resampled OTU subsets under the 90% of the minimum sequencing depth for further analysis.

For bacterial 16S rRNA genes, the representative sequences were annotated against the SILVA database (http://www.arb-silva.de); for fungal ITS sequences, the representative sequences were annotated against the UNITE database (https://unite.ut.ee/). OTU-level alpha diversity indices, such as Chao1 richness estimator, Shannon diversity index, and Simpson index, were calculated using the OTU table in QIIME. Beta diversity analysis was performed to investigate the structural variation of microbial communities across samples using UniFrac distance metrics (Lozupone and Knight, 2005) and visualized via principal coordinate analysis (PCoA). A comparison of overall microbial distribution among samples was conducted on the relative abundances of phyla or genus using OTUs based on taxonomy. All the above analyses, including the community heatmap at the genus level, were performed using R software (v.3.5.3).

### Transcriptome analysis

Root samples 15 d post the application of biocontrol agents at the concentration of 1:150 were collected, with three independent biological replicates per sample. Total RNA from each sample was extracted using the Total RNA Extraction Kit (AM1561, Ambion), following the manufacturer’s instructions. The quality of RNA was examined using Agilent Bioanalyzer 2100 system. A total of 1.5 μg of RNA per sample was used as the input materials to create sequencing libraries using NEBNext Ultra Directional RNA Library Prep kit (NEB, Beverly, MA, USA), and sequencing was performed at Biotree Technology Co. Ltd. (Shanghai, China) on an Illumina Novaseq 6000 platform. Low-quality reads and adapter sequences were removed from raw reads through fastp software before assembly. The sequencing clean reads were remapped to the reference sequence using Hisat2 v2.2.1 software.

DESeq2 v1.42.0 software was used to compute the differential expression post the application of biocontrol agents compared to tomato plants without treatment. Genes with a minimal 2-fold difference in expression (|log2 (fold change)| ≥ 1) and *P_adj_ ≤* 0.05 were considered as differential expressed genes (DEGs), and DEGs were used for the following KEGG(Kyoto Encyclopedia of Genes and Genomes, http://www.genome.jp/kegg/) pathway-enrichment analysis with clusterProfiler v4.8.1 software. Pathways with a corrected *p* value of <0.05 are considered to be significantly enriched.

### Metabolome analysis

Root samples were harvested at 15 d post the application of biocontrol agents at the concentration of 1:150, and non-treated samples were used as control. Three biological replicates for each treatment were evaluated. The samples (20 mg ± 1 mg) were lyophilized, mixed with beads and 1000 μL of extraction solution (MeOH: ACN: H_2_O, 2:2:1 (v/v)). The extraction solution contains deuterated internal standards. The mixed solution was vortexed for 30 s. Then the mixed samples were homogenized (35 Hz, 4 min) and sonicated for 5 min in 4°C water bath, the step repeat for three times. The samples were incubated for 1 h at -40°C to precipitate proteins. Then the samples were centrifuged at 12000 rpm for 15 min at 4°C. Transfer 400 μL of liquid to the well of a protein precipitation plate. Place the plate on the manifold. Apply vacuum, 6 psi, 120 s. Take plate from the positive pressure device for analysis. The quality control (QC) of sample was prepared by mixing an equal aliquot of the supernatant of samples.

Metabolites were then analyzed through untargeted LC-MS/MS technology. Briefly, extraction of samples (2 μL) was injected into an Ultra Performance Liquid Chromatography (UPLC, Vanquish, Thermo Fisher Scientific) equipped with a tandem mass spectrometry (MS/MS, Orbitrap Exploris 120) bound with Waters ACQUITY UPLC BEH Amide column (2.1 mm × 100 mm, 1.7 μm). For polar metabolites, the mobile phase consisted of water (25 mmol/L ammonium acetate and 25 mmol/L ammonia water, A) and acetonitrile (B). For non-polar metabolites, the mobile phase consisted of water (0.01% acetic acid, A) and isopropyl alcohol: acetonitrile (1:1, v/v, B).

The detailed parameters of the mass spectrum are as follows: sheath gas flow rate, 50 Arb; aux gas flow rate, 15 Arb; capillary temperature, 320°C; full ms resolution, 60000; MS/MS resolution, 15000; collision energy, SNCE 20/30/40; spray voltage, 3.8 kV (positive) or -3.4 kV (negative). The raw data were processed using ProteoWizard software. The metabolites were identified by matching the secondary spectral information with the BiotreeDB (V3.0) database, and quantified by integrating peak areas. The multivariate analysis of identified metabolites was performed by Principal Component Analysis (PCA). 

### Transcriptome and metabolome correlation analysis

Differentially expressed genes (DEGs) mapped to the “Plant-pathogen interaction” pathway and differentially accumulated metabolites (DAMs) in pathways related to plant immunity were selected for correlation analysis. The correlation analysis was performed using Pearson’s correlation coefficient in the R language corr.test package to calculate the correlation coefficient and the *p*-value. The results were visualized by R language package.

## Results

### *Bacillus velezensis* or *P. fluorescens* had significant inhibitory effect on the growth of *R. solanacearum*

**Figure 1:**
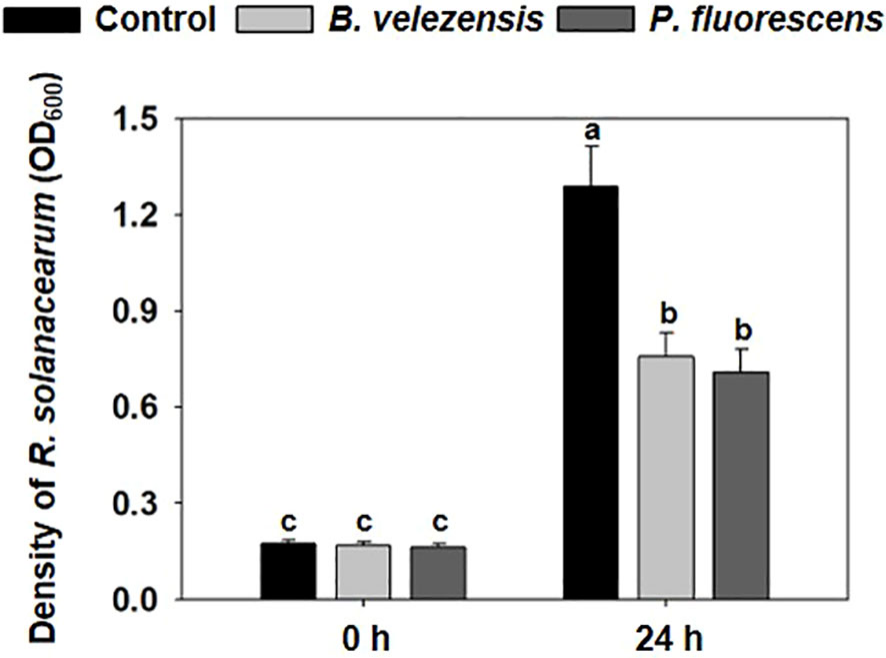
Effect of *B. velezensis* or *P. fluorescens* on the growth of *R. solanacearum in vitro*. The densities of *R. solanacearum* grown alone in NB medium or inoculated with the fermentation broth of biocontrol agents after 0 h or 24 h. The optical density (OD_600_ (nm)) was measured using a spectrophotometer. all treatments were measured with 12 biological replicates. Different letters indicate significant differences (*P*<0.05) according to Student’s *t* test after arcsine transformation.

We tested the inhibitory effect of biocontrol agents on the growth of *R. solanacearum in vitro*. When *R. solanacearum* was cultured alone in NB medium for 24 h, its density reached to OD_600_ = 1.288. After cultured with fermentation broth of *B. velezensis* or *P. fluorescens*, the density of *R. solanacearum* significantly decreased to 0.755 or 0.707 (**Figure 1**), respectively, indicating the biocontrol potential of these beneficial bacteria against the *R. solanacearum* pathogen. There was no significant difference between these two kinds of fermentation broth in inhibiting the growth of *R. solanacearum*.

### *Bacillus velezensis* or *P. fluorescens* effectively control tomato bacterial wilt and promote plant growth in the field

**Figure 2:**
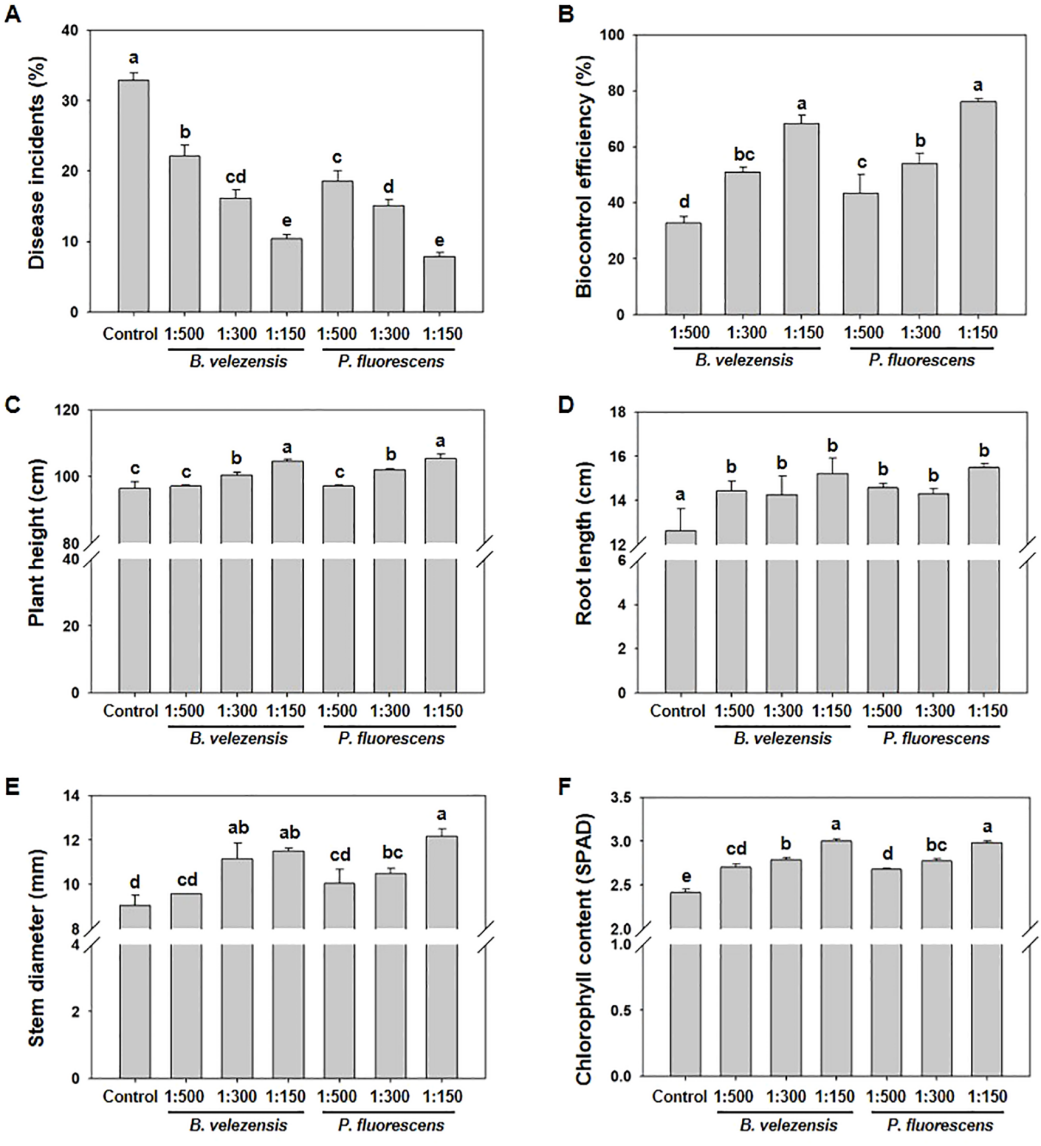
Treatment with *B. velezensis* or *P. fluorescens* enhances resistance to tomato bacterial wilt and promotes plant growth. **(A)** Disease incidents of tomato bacterial wilt after the application of biocontrol agents with different concentration (1:500, 1:300, 1:150). **(B)** Biocontrol efficiency of biocontrol agents with different concentration (1:500, 1:300, 1:150) against tomato bacterial wilt. **(C–F)** Plant height **(C)**, root length **(D)**, stem diameter **(E)**, and chlorophyll content **(F)** of tomato plants treated with or without biocontrol agents of different concentration (1:500, 1:300, 1:150). All data were measured at 15 d after the application of biocontrol agents. Different letters indicate significant differences (*P*<0.05) according to Student’s *t* test after arcsine transformation.

In order to explore the effect of controlling tomato bacterial wilt in the field, *B. velezensis* (2×10^8^ CFU/g) or *P. fluorescens* (5×10^8^ CFU/g) were diluted to 1:500, 1:300 and 1:150, and applied to tomato plants before the onset of tomato bacterial wilt and at the flowering stage, and then the control effect of disease was investigated at 15 d after the treatment. The results of the field test showed that all treatments significantly reduced disease severity of tomato bacterial wilt (**Figure 2A**). Compared with non-treated control plants, the biocontrol efficacy of *B. velezensis* or *P. fluorescens* of 1:500 reached 32.69% and 43.42%, respectively. With the increase of concentration, the biocontrol efficiency was significantly improved, with *P. fluorescens* of 1:150 reached 76.08% and *B. velezensis* of 1:150 reached 68.40% (**Figure 2B**).

Correspondingly, the application of these biocontrol agents significantly promoted the growth of tomato plants compared to control plants. The promotion effect was also gradually enhanced with the increase of concentration. When the dilution concentration of *P. fluorescens* reached 1:150, the average plant height, root length and stem thickness significantly increased from 96.47 cm, 13.14 cm and 9.06 mm to 105.49 cm, 15.48 cm and 11.75 mm, respectively. Meanwhile, *B. velezensis* of 1:150 showed similar effect on plant growth, which increased plant height, root length and stem diameter to 104.60 cm, 15.21 cm and 11.50 mm, respectively (**Figures 2C–E**).

Pathogen infection often leads to a decrease in photosynthetic assimilates production, which can be analyzed by monitoring chlorophyll content (SPAD value) (Singh and Siddiqui, 2015). Studies have shown that the chlorophyll content of plant leaves can be significantly increased after inoculation with biocontrol bacteria (Xue et al., 2016; Jamil et al., 2021). Compared with control plants, *B. velezensis* or *P. fluorescens* of 1:150 increased chlorophyll content from 2.41 SPAD to 2.98 SPAD or 3.00 SPAD, respectively, which is also concentration-dependent (**Figure 2F**), playing an important role in increasing the efficiency of photosynthesis in plants.

Therefore, these results indicate that biocontrol bacteria, such as *B. velezensis* and *P. fluorescens*, significantly improve the ability of tomato plants against bacterial wilt, thus promote their growth and development.

### The composition of bacterial communities in rhizosphere soil changed significantly by biocontrol agents

**Figure 3:**
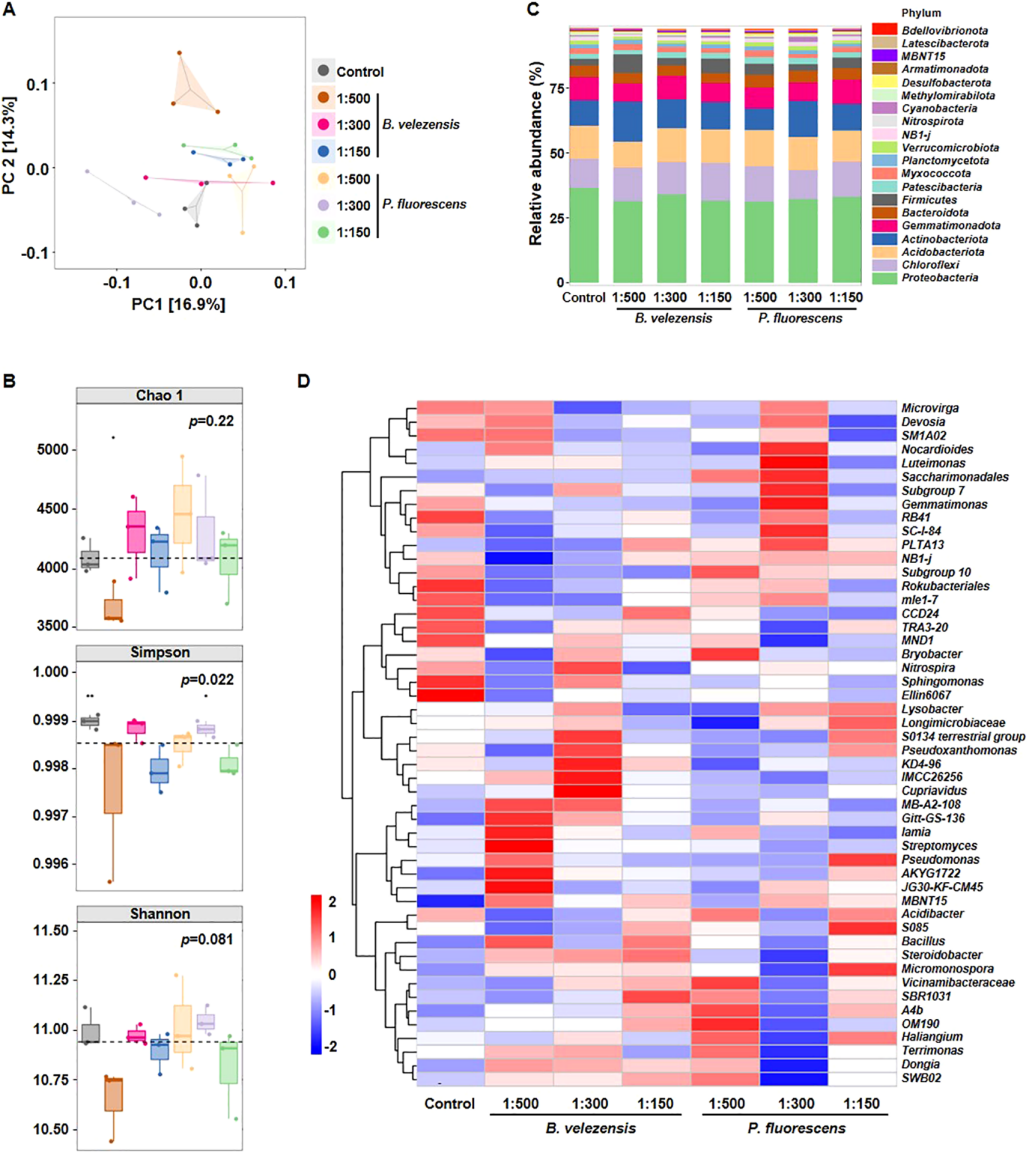
Diversity differences and community compositions of bacterial communities after the application of *B. velezensis* or *P. fluorescens*. **(A, B)** PCoA analysis **(A)**, Chao 1 index, Simpson index, and Shannon index **(B)** showing clustering relationship of bacterial communities among different samples. Samples of different groups are color-coded. **(C)** Community composition of bacterial communities in different samples at the phylum level. **(D)** Heatmap showing the relative abundance of dominant bacterial taxon (top 50) at the genus level among different samples.

Current studies mainly focus on the effect of bacterial wilt on the community composition in rhizosphere soil of multiple economically important crops, such as tobacco, pepper, and potato (Kong et al., 2022; Zheng et al., 2022). However, how this effect is influenced by biocontrol agents remains unclear. The diversity of bacterial communities in the rhizosphere soil of infected tomato plants treated with or without biocontrol agents was analyzed, and more than 60,000 high-quality 16S rRNA sequences were obtained for each sample. Principal coordinates analysis (PCoA) was conducted to confirm the differences among samples (**Figure 3A**). The degree of explanation of principal coordinate 1 (PC1) and PC2 for the soil sample differences was 16.9% and 14.3%, respectively. The results revealed that bacterial communities of different treatments were clustered, suggesting that *B. velezensis* or *P. fluorescens* had a great impact on the composition of bacterial communities.

Alpha diversity of bacterial communities was evaluated to more comprehensively understand the effects of these biocontrol agents on community structure. The Chao 1 results showed that the richness of bacterial species was not significantly changed among all treatments. By contrast, the Simpson index had a significant difference (*p*<0.05), and the Shannon diversity was also different although not significantly (*p*=0.081), indicating that *B. velezensis* or *P. fluorescens* affected the bacterial diversity in the rhizosphere of infected tomato (**Figure 3B**). Given that *B. velezensis* or *P. fluorescens* of 1:150 demonstrated a more pronounced improvement in plant growth and disease resistance, we further directly compared the bacterial community composition between the control and groups treated with biocontrol agents of high concentration. Similarly, no significant differences were observed in Chao 1 and Shannon indices, whereas the Simpson index was lower in groups treated with *B. velezensis* or *P. fluorescens* of 1:150, indicating a significantly reduced bacterial diversity (*p*=0.05, **Supplementary Figure S1**).

At the phylum level, *Proteobacteria*, *Chloroflexi*, *Acidobacteriota*, *Actinobacteriota*, and *Gemmatimonadota* were the dominant bacteria in all treatments, accounting for more than 75% of the total amount of bacterial. The community composition of these treatments was similar, but the relative abundance of each phylum did differ in the rhizosphere soil samples. Specifically, the relative abundance of the *Proteobacteria*, which contains the pathogenic species of bacterial wilt disease, was significantly decreased to 31.4%-34.1% in the groups treated with *B. velezensis* or *P. fluorescens*, compared to 36.7% in the group without biocontrol agent treatment (**Figure 3C**).

According to the relative abundance of each sample at the genus level, the top 50 taxon were selected to generate a bacterial community heatmap (**Figure 3D**). Compared with control, in which *Ellin6067*, *Sphingomonas*, *MND1*, *TRA3-20*, *CCD24*, *mle1–7* and *Rokubacteriales* were more abundant, the effects of different concentrations and types of biocontrol agents on the composition of the bacterial community were not exactly the same. For example, the genera *Gitt-GS-136*, *Iamia*, *Streptomyces*, *AKYG1722*, and *JG30-KF-CM45* were significantly enriched in groups treated with *B. velezensis* of 1:500, while the genera *S0134 terrestrial group*, *Pseudoxanthomonas*, *KD4-96*, *IMCC26256*, and *Cupriavidus* were significantly more abundant in groups treated with *B. velezensis* of 1:300. In contrast, *Nocardioides*, *Luteimonas*, *Saccharimonadales*, and *Gemmatimonas* were significantly enriched in groups treated with *P. fluorescens* of 1:300. These results suggest that the effects of different biocontrol agents on rhizosphere bacterial may not be identical, even though they all reduced the severity of tomato bacterial wilt and promoted plant growth.

### Biocontrol agents had less effect on fungal community composition in rhizosphere soil of infected tomato plants

**Figure 4:**
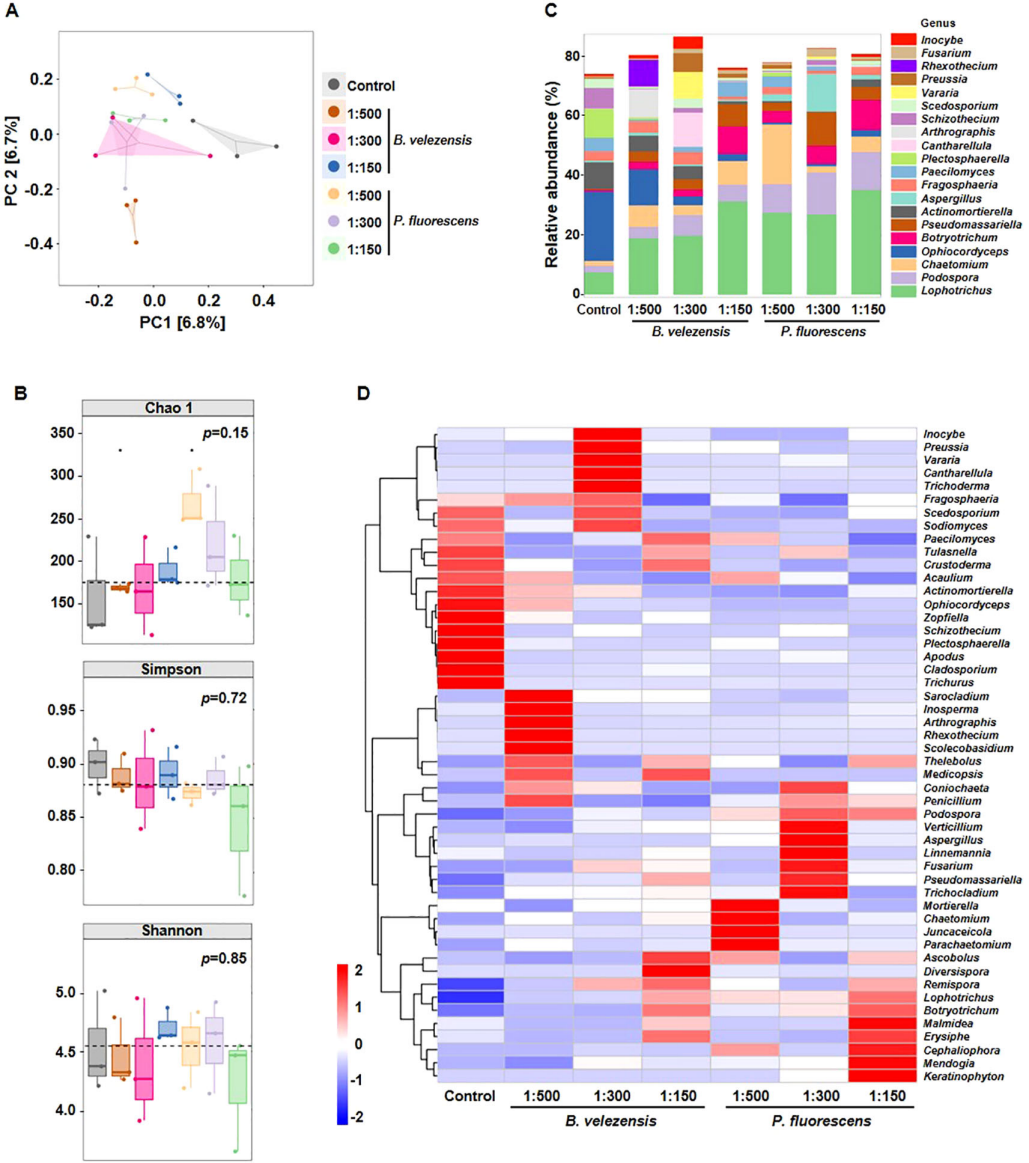
Diversity differences and community compositions of fungal communities after the application of *B. velezensis* or *P. fluorescens*. **(A, B)** PCoA analysis **(A)**, Chao 1 index, Simpson index, and Shannon index **(B)** showing clustering relationship of fungal communities among different samples. Samples of different groups are color-coded. **(C)** Community composition of fungal communities in different samples at the genus level. **(D)** Heatmap showing the relative abundance of dominant fungal taxon (top 50) at the genus level among different samples.

The involvement of soil fungus in antagonistic interaction with soil-borne pathogens have been comprehensively and precisely illustrated by previous studies (Siegel-Hertz et al., 2018; Zhou et al., 2019; Zhang et al., 2022), thus we also examined the effect of biocontrol agents on fungal community structure in rhizosphere soil of infected tomato plants. After quality control, more than 80,000 high-quality fungal ITS sequences were obtained for each sample. Concerning fungal communities, the degree of explanation of PC1 and PC2 was only 6.7% and 6.8% (**Figure 4A**), indicating that sample discrimination was not obvious. Similarly, no obvious differences in species richness according to Chao 1 indices or species diversity according to Simpson and Shannon indices were observed among these samples (**Figure 4B**). Furthermore, no significant differences were observed in Chao1, Simpson, and Shannon indices between the control and groups treated with the highest concentration (**Supplementary Figure S2**), suggesting that the effect of these biocontrol agents on fungal community structure in rhizosphere soil was less than that on bacterial community.

However, the relative abundance of unique genera was different among samples. The application of biocontrol agents led to a remarkable enrichment of potentially beneficial genera (e.g., *Lophotrichus*, *Botryotrichum*), in stark contrast to the depletion of fungal pathogens (e.g., *Ophiocordyceps*, *Plectosphaerella*) (**Figure 4C**) (Hinton and Parry, 1993; Kobmoo et al., 2015; Tang et al., 2023; Wang et al., 2024), emphasizing the potential significance of these biocontrol agents in preserving the health of tomato plants through specific fungal genera.

Moreover, different concentrations of biocontrol agents produce distinct dominant species, and fungal species affected by *B. velezensis* and *P. fluorescens* were also different (**Figure 4D**). For example, *Ascobolus* and *Diversispora* were enriched in the group treated with *B. velezensis* of 1:150, while *Mendogia* and *Keratinophyton* were dominant in the group treated with *P. fluorescens* of 1:150.

### Biocontrol agents lead to transcriptional reprogramming in tomato infected with bacterial wilt

Considering that the high concentration (1:150) of biocontrol agents has a more obvious improvement in biocontrol efficiency and plant growth (**Figure 2**), transcriptome analysis was conducted using roots of infected tomato with or without biocontrol agents (1:150), to understand their underlying molecular mechanisms against tomato bacterial wilt. The number of clean reads for each sample ranged from 45.58 million to 52.50 million. Each library generated >6 Gb of clean bases. The percentage of Q20 was >97% and the percentage of Q30 was >93%.

**Figure 5:**
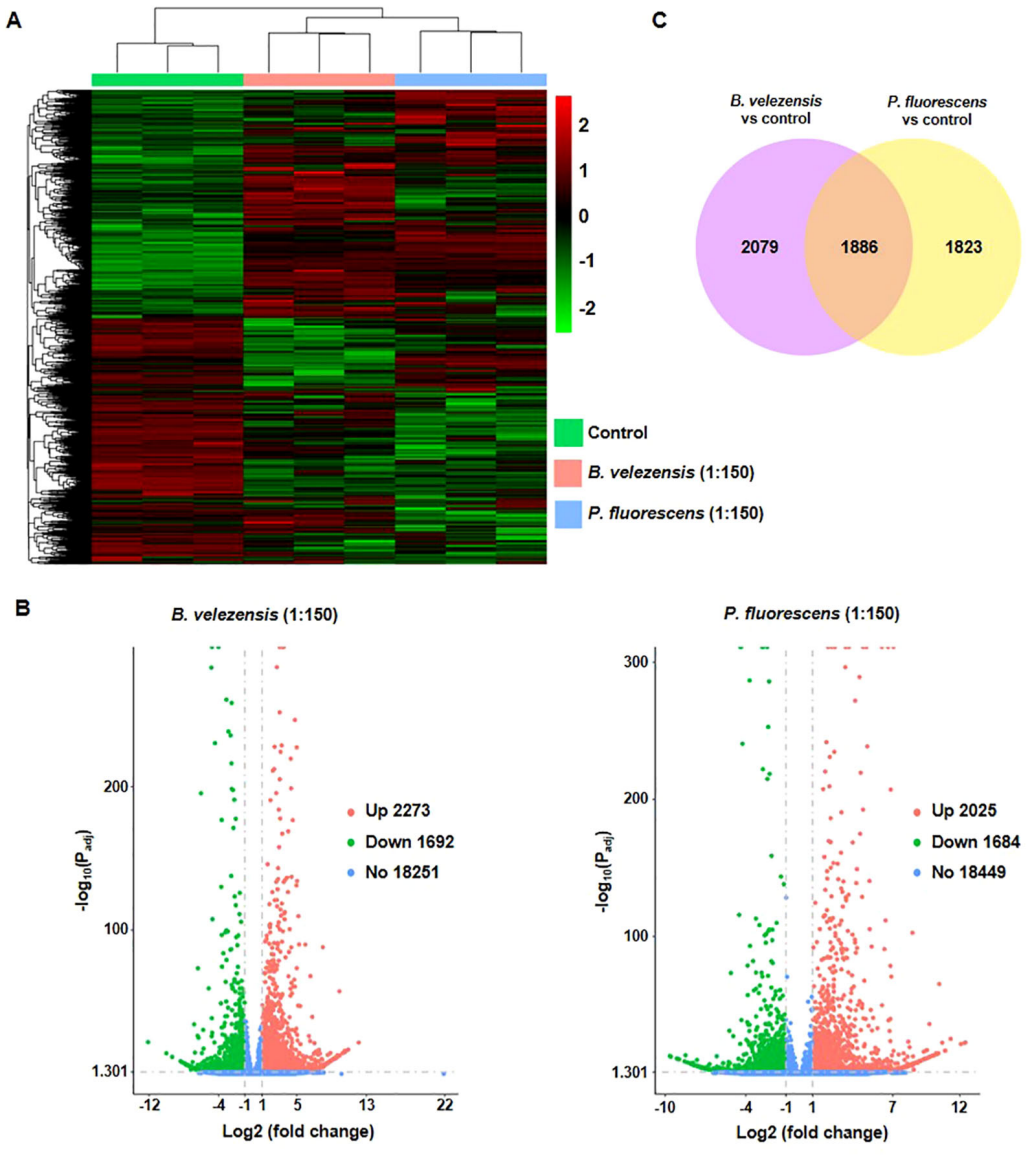
Identification of differentially expressed genes (DEGs) in infected tomato plants treated with biocontrol agents. **(A)** Heatmap showing that the transcriptome file changed significantly in groups treated with biocontrol agents of 1:150. **(B)** The volcano diagram showing DEGs in tomato plants treated with biocontrol agents of 1:150. Red, green, and blue dots represent the up-regulated (Up), down-regulated (Down), and no response (No) genes, respectively. Numbers of genes were shown in the figure. **(C)** The Venn diagram analysis showed the overlapped DEGs between groups treated by different biocontrol agents.

*Bacillus velezensis* or *P. fluorescens* with high concentration induced intense transcriptome reprogramming in tomato plants (**Figure 5A**). Results showed that *B. velezensis* induced 3965 differentially expressed genes (DEGs), including 2273 up- and 1692 down-regulated DEGs (**Figure 5B**; **Supplementary Table S1**). In contrast, *P. fluorescens* induced 3709 DEGs, including 2025 up- and 1684 down-regulated genes (**Figure 5B**; **Supplementary Table S2**). Among them, 1886 DEGs are overlapped between different treatment, and others are biocontrol agents-specific (**Figure 5C**).

To investigate the biological processes and molecular functions that changes after the application of biocontrol agents, DEGs were mapped to the KEGG pathway database simultaneously under the criteria |log_2_ (fold change) | ≥ 1 and *P_adj_* ≤ 0.05. The top 20 terms were selected to draw scatter plots for display.

**Figure 6:**
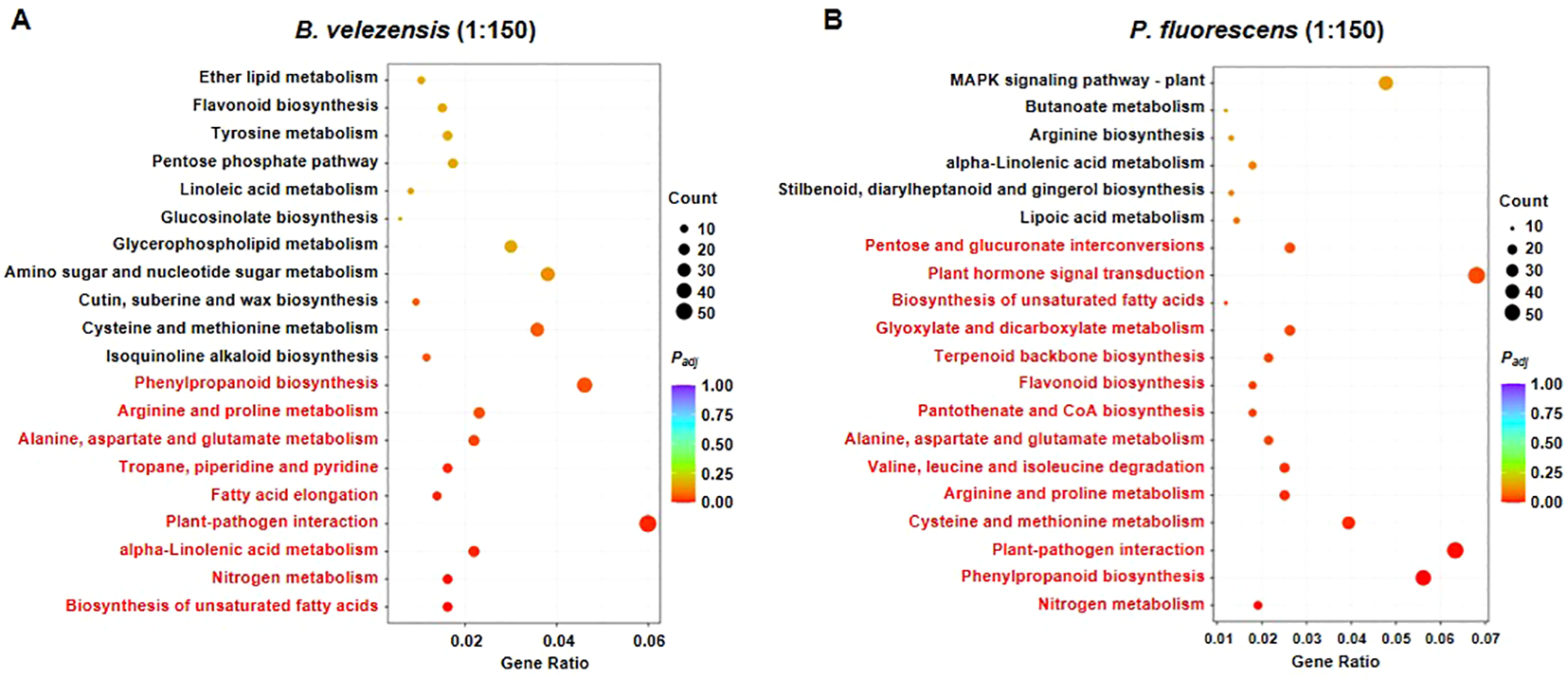
KEGG pathway enriched in the group treated with *B. velezensis* **(A)** or *P. fluorescens* **(B)**. Gene Ration represents the ration of numbers of differentially expressed genes annotated in the pathway term to the numbers of all genes annotated in the same pathway. Top 20 pathway terms enriched are displayed in the figure. The bar color from red to purple represents the significance of the enrichment, and significantly enriched KEGG pathways are highlighted in red.

For the group treated with *B. velezensis* of 1:150, many biosynthesis and metabolism processes including “Phenylpropanoid biosynthesis”, “Arginine and proline metabolism”, “Alanine, aspartate and glutamate metabolism”, “alpha-Linolenic acid metabolism”, “Nitrogen metabolism” and “Biosynthesis of unsaturated fatty acids” were significantly enriched (**Figure 6A**), many of which play important roles in plant immunity (Irani et al., 2019; Zhang et al., 2022; Xiao et al., 2023; Zhu et al., 2024). In addition, the “Plant-pathogen interaction” pathway was also significantly upregulated in the sample (**Figure 6A**). These results suggest that high concentrations of this biocontrol agent activate a variety of metabolic and signaling pathways in tomato plants to help them increase the resistance against *R. solanacearum*, which is consistent with its significant improvement in disease resistance and plant growth (**Figure 2**).

In contrast, in addition to the same pathways activated by *B. velezensis* of 1:150, some other metabolism processes such as “Flavonoid biosynthesis” which are very important for plant defense against pathogens (Treutter, 2005), were also enriched in the group treated with *P. fluorescens* of high concentration (**Figure 6B**). Notably, “Plant hormone signal transduction” was activated by this treatment (**Figure 6B**). Previous studies revealed that salicylic acid (SA) and Jasmonate (JA) are major defense-related phytohormones, other phytohormones such as ethylene (ET), auxin, gibberellins (GAs), abscisic acid (ABA), brassinosteroids (BRs), and cytokinins (CKs) are also involved in defense responses (Dermastia, 2019). We suppose that plant hormone signal transduction may be specifically activated by this beneficial microorganism, and thus regulate plant disease resistance coordinating with biosynthesis/metabolism and plant-pathogen interaction pathways, although it is not known which plant hormone plays a major role in this process.

Overall, the application of these biocontrol agents led to dramatic transcriptome reprogramming in tomatoes infected with bacterial wilt. Different types of biocontrol agents may improve plant resistance by regulating specific biosynthesis/metabolism processes or signaling pathways to maintain the balance between plant growth and biological stress.

### Specific metabolites play an important role in the regulation of tomato bacterial wilt by biocontrol agents

**Figure 7:**
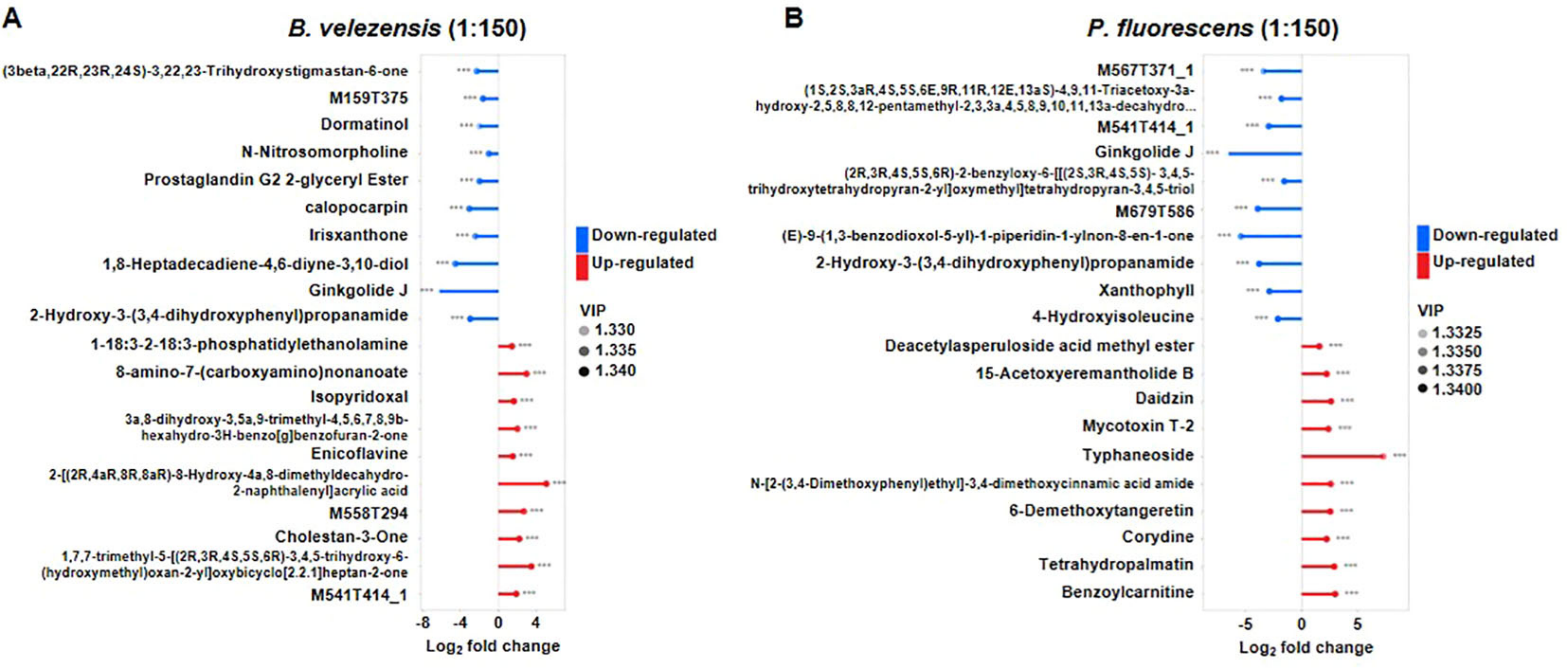
Metabolite changed in the group treated with *B. velezensis* **(A)** or *P. fluorescens* **(B)**. Metabolites with *p* value of <0.05 and the variable importance in project (VIP) > 1 were considered to be differentially accumulated metabolites (DAMs). Moreover, the databases KEGG and PubChem (https://pubchem.ncbi.nlm.nih.gov/) were used to search the differential metabolic pathways.

To gain the overview of metabolites affected by these biocontrol agents in infected tomato plants, an untargeted metabolomics by LC-MS/MS was performed to evaluated metabolite profiles in detail. Similarly, the roots of infected tomatoes treated with high concentrations (1:150) of *B. velezensis* or *P. fluorescens* were analyzed. Metabolites with *p* value of <0.05 and the variable importance in project (VIP) > 1 were considered to be differentially accumulated metabolites (DAMs) (**Supplementary Tables S3**, **S4**), and the top 10 DAMs significantly up-regulated and down-regulated were selected for display (**Figure 7**).

Both of biocontrol agents resulted in a reduction of Ginkgolide J and the 2-Hydroxy-3-(3,4-dihydroxyphenyl)propenamide, whose superoxide scavenging effect contributes to the antioxidant properties (Scholtyssek et al., 1997). Metabolite profiles revealed that specific metabolites were enriched or reduced after the application of these biocontrol agents. Particularly, 1-18:3-2-18:3-phosphatidylethanolamine, 8-amino-7-(carboxyamino)nonanoate, isopyridoxal and cholestan-3-one were significantly more abundant in the group treated with *B. velezensis*, while deacetylasperuloside acid methyl ester, daidzin, mycotoxin T-2 and typhaneoside et al. were enriched in the group treated with *P. fluorescens* (**Figure 7**). These results suggested that different biocontrol agents may induce the production of specific metabolites in infected tomato plants to resist bacterial wilt.

Metabolites with *p* value of <0.05 and the variable importance in project (VIP) > 1 were considered to be differentially accumulated metabolites (DAMs), and the top 10 DAMs significantly up-regulated and down-regulated were selected for display. Red and blue terms represent the up- and down-regulated metabolites, respectively.

Key pathways that are most relevant to the metabolite differences were analyzed, and results showed that “Pantothenate and CoA biosynthesis” has the highest correlation with different metabolites in the group treated with *B. velezensis* of 1:150, while “Phenylalanine metabolism” was the highest when treated with *P. fluorescens* of high concentration (**Supplementary Figure S3**), further clarifying the differences in the effects of these two biocontrol agents on the metabolism of infected tomato plants.

### Correlation analysis of transcriptome and metabolome data

To further reveal the differences between each biocontrol bacteria as well as their impact on plant innate immunity in greater depth, we analyzed the correlation between DEGs mapped to the “Plant-pathogen interaction” pathway and DAMs in the metabolic pathways that may be involved in plant immunity. There are 51 and 53 DEGs mapped to the “Plant-pathogen interaction” pathway in the group treated with *B. velezensis* or *P. fluorescens* of 1:150, respectively (**Supplementary Tables S5**, **S6**). Among pathways significantly relevant to the metabolite differences, “Pantothenate and CoA biosynthesis”, “Glycine, serine and threonine metabolism”, and “Phenylalanine metabolism” have been previously reported to be involved in the defense-related responses (**Supplementary Figure S3**) (Ma et al., 2020; Wu et al., 2023; Li et al., 2025). Therefore, 14 and 12 DAMs mapped to these metabolic pathways in the group treated with *B. velezensis* or *P. fluorescens*, respectively, were selected for correlation analysis (**Supplementary Tables S7**, **S8**).

**Figure 8:**
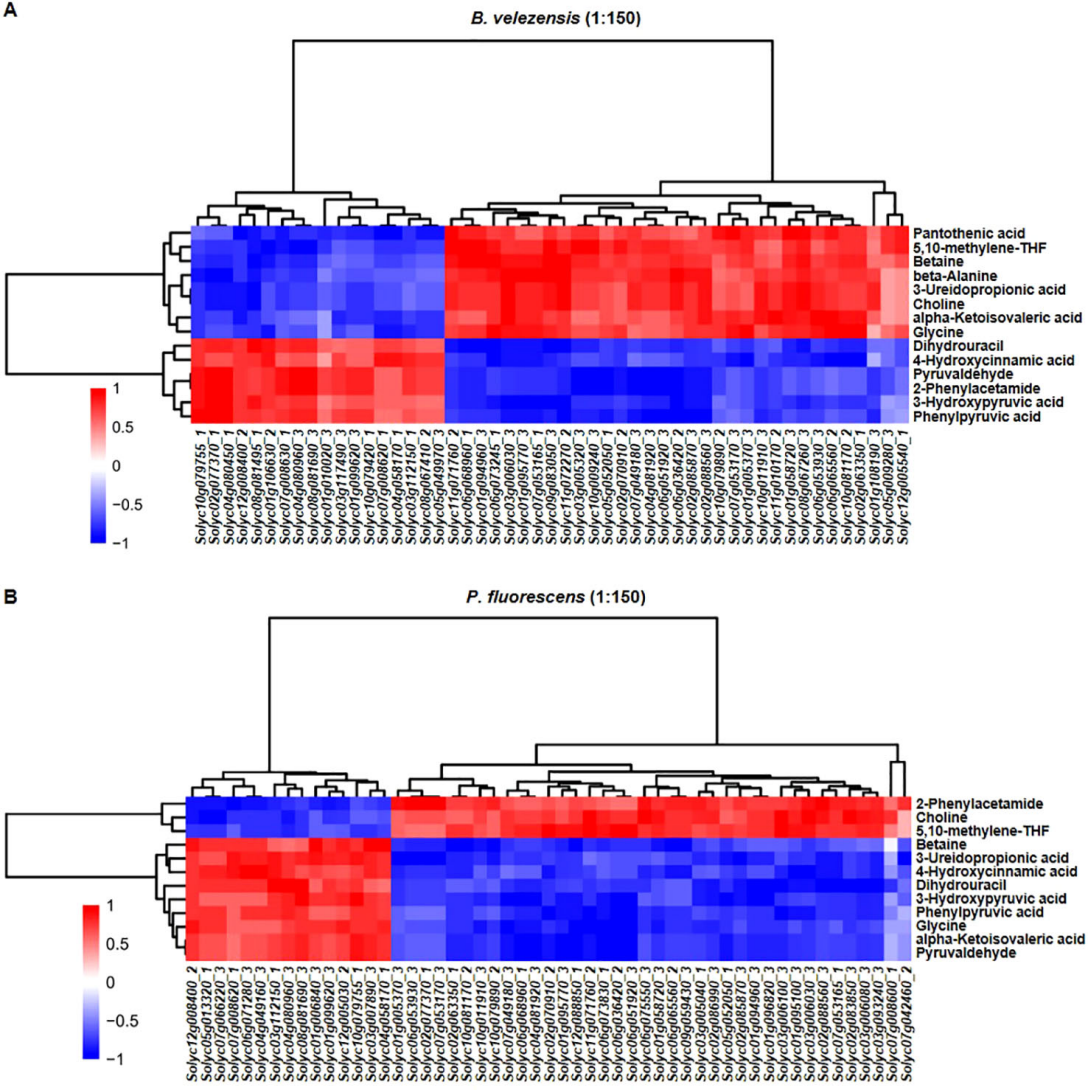
Overview of the correlation between DEGs and DAMs. Correlation analysis of DEGs mapped to the “Plant-pathogen interaction” pathway and DAMs in the metabolic pathways related to plant immunity in the group treated with *B. velezensis* [51 transcripts and 14 metabolites, **(A)**] or *P. fluorescens* [53 transcripts and 12 metabolites, **(B)**]. The vertical direction represents the DAMs cluster, and the horizontal direction represents the DEGs cluster. Red indicates positive correlation, and blue indicates negative correlation.

The results show that most of genes related to plant-pathogen interaction were positively correlated with pantothenic acid, 5, 10-methylene-THF, betaine, beta-alanine, 3-ureidopropionic acid, choline, alpha-ketoisovaleric acid and glycine in the group treated with *B. velezensis*. In contrast, except for 5, 10-methylene-THF and choline, most genes were only positively correlated with 2-phenylacetamide when treated with *P. fluorescens* (**Figure 8**). Although the “Plant-pathogen interaction” pathway was up-regulated in both groups, they might promote the production of different immune-related metabolites to promote tomato-*R. solanacearum* interaction.

## Discussion

Biological control has become an increasingly important means to inhibit plant diseases due to its green and efficient advantages. Our understanding of the ways in which biocontrol agents protect plants from disease has developed considerably in recent years, which is crucial to the development of biocontrol strategies for plant diseases (O’Brien, 2017). This study revealed that biocontrol agents, including *B. velezensis* and *P. fluorescens*, significantly inhibited the growth of *R. solanacearum in vitro* (**Figure 1**). At the same time, tomato bacterial wilt that is caused by *R. solanacearum* was efficiently controlled by the biocontrol agents, promoting the growth of tomato plants in the field (**Figure 2**), which makes these biocontrol agents an important strategy to resist tomato bacterial wilt.

We found that the composition of bacterial community in rhizosphere soil changed more significantly than that of fungal community after the application of biocontrol agents, with more abundant beneficial microorganisms and reduced harmful microorganisms (**Figures 3**, **4**). In order to further investigate how these biocontrol agents improve the disease resistance of plants and whether their regulatory mechanisms are specific, we analyzed the transcriptome and metabolome changes of infected tomato roots. Results showed that *B. velezensis* activated the synthetic/metabolic pathway and plant-pathogen interaction pathway in infected tomato plants, while *P. fluorescens* also activated some other pathways such as plant hormone signal transduction (**Figures 5**, **6**). Among them, secondary metabolites derived from biosynthetic pathways such as those for flavonoids and phenylpropanoid are essential in plant innate immunity and response to environmental stresses (Dixon, 2001; Pang et al., 2021). Further correlation analysis of transcriptome and metabolome data revealed the differences between each biocontrol bacteria as well as their impact on plant innate immunity (**Figure 8**).

Hormone-regulated genes play an essential role in regulating plant response to biotic and abiotic stress. In general, plant defense responses include SA-dependent defenses acting on biotrophs and JA/ET-dependent responses acting on necrotrophs (Kim et al., 2022). Some beneficial microbes could trigger systemic resistance by regulating SA- and JA/ET-mediated defense genes, such as pathogenesis-related (*PR*) genes (e.g., *PR1*, *PR2*) and ethylene-responsive factors (*ERF*s), strengthening plant immunity against tomato bacterial wilt (Le et al., 2021; Wei et al., 2024b). Our findings demonstrate that *P. fluorescens* activates plant hormone signal transduction, whereas *B. velezensis* does not. Further validation is required to determine which specific plant hormone signaling pathway and its associated genes are activated by *P. fluorescens* to enhance resistance against tomato bacterial wilt.

Previous studies have shown that *B. velezensis* could produce antibacterial substances such as lipopeptides and polyketides at ecological sites to exert antagonistic effects, inhibiting motility traits of *R. solanacearum* and damaging the cell wall (Villegas-Escobar et al., 2018; Wang et al., 2023). In contrast, the 2,4-diacetylphloroglucinol (DAPG) from the crude metabolites of *P. fluorescens* has the antimicrobial potential against *R. solanacearum* (Suresh et al., 2021). Specific reactive oxygen species and hormone signaling cascades induced by *P. fluorescens* may also contribute to its role as beneficial microorganism (Zhou et al., 2016). We speculate that the differences in the regulatory mechanisms of these two kinds of biocontrol agents may be related to the substances they produce, although their biocontrol efficiency are similar. These biocontrol agents may recruit specific microorganisms in rhizosphere soil by producing different metabolites, and activated specific immune response through different pathways, thus protect tomato from bacterial wilt. Our study provides new insight into the specific mechanisms of different biocontrol agents against *R. solanacearum*, and establishes a theoretical basis for developing compound biocontrol agents.

## Data Availability

The original contributions presented in the study are included in the article/**Supplementary Material**, further inquiries can be directed to the corresponding authors.
