# Supplementary material for: Multi-omics analysis reveals the specific role of biocontrol reagents against tomato bacterial wilt
Source: Front Plant Sci. 2025 Jul 14;16:1620460. doi: 10.3389/fpls.2025.1620460 (PMC12301371; doi:10.3389/fpls.2025.1620460)
Supplement: Supplementary file 9 [file Presentation1.pptx]

## Slide 1
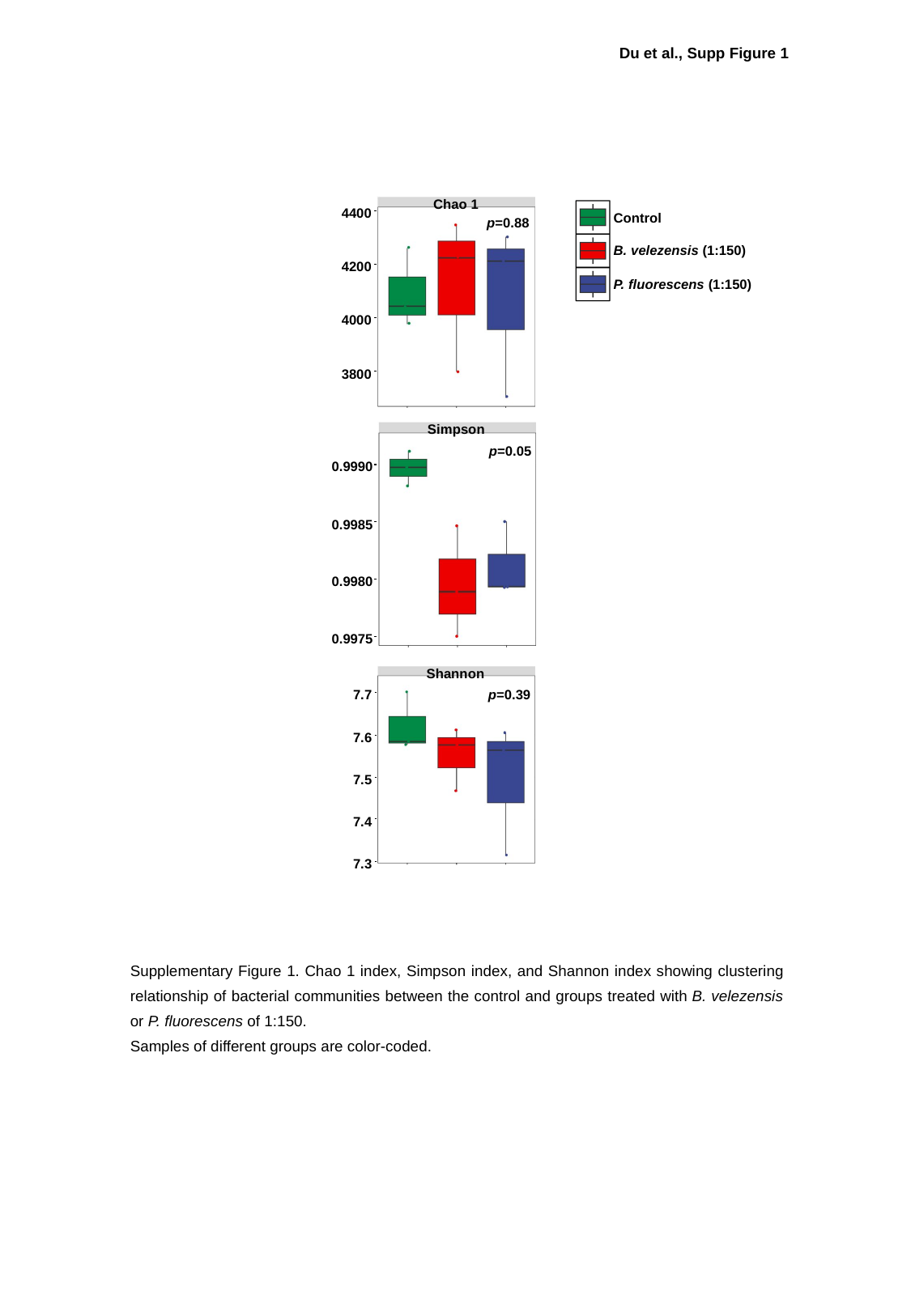

Du et al., Supp Figure 1
Chao 1
4400
Control
p=0.88
B. velezensis (1:150)
4200
P. fluorescens (1:150)
4000
3800
Simpson
p=0.05
0.9990
0.9985
0.9980
0.9975
Shannon
7.7
p=0.39
7.6
7.5
7.4
7.3
Supplementary Figure 1. Chao 1 index, Simpson index, and Shannon index showing clustering relationship of bacterial communities between the control and groups treated with B. velezensis or P. fluorescens of 1:150.
Samples of different groups are color-coded.

## Slide 2
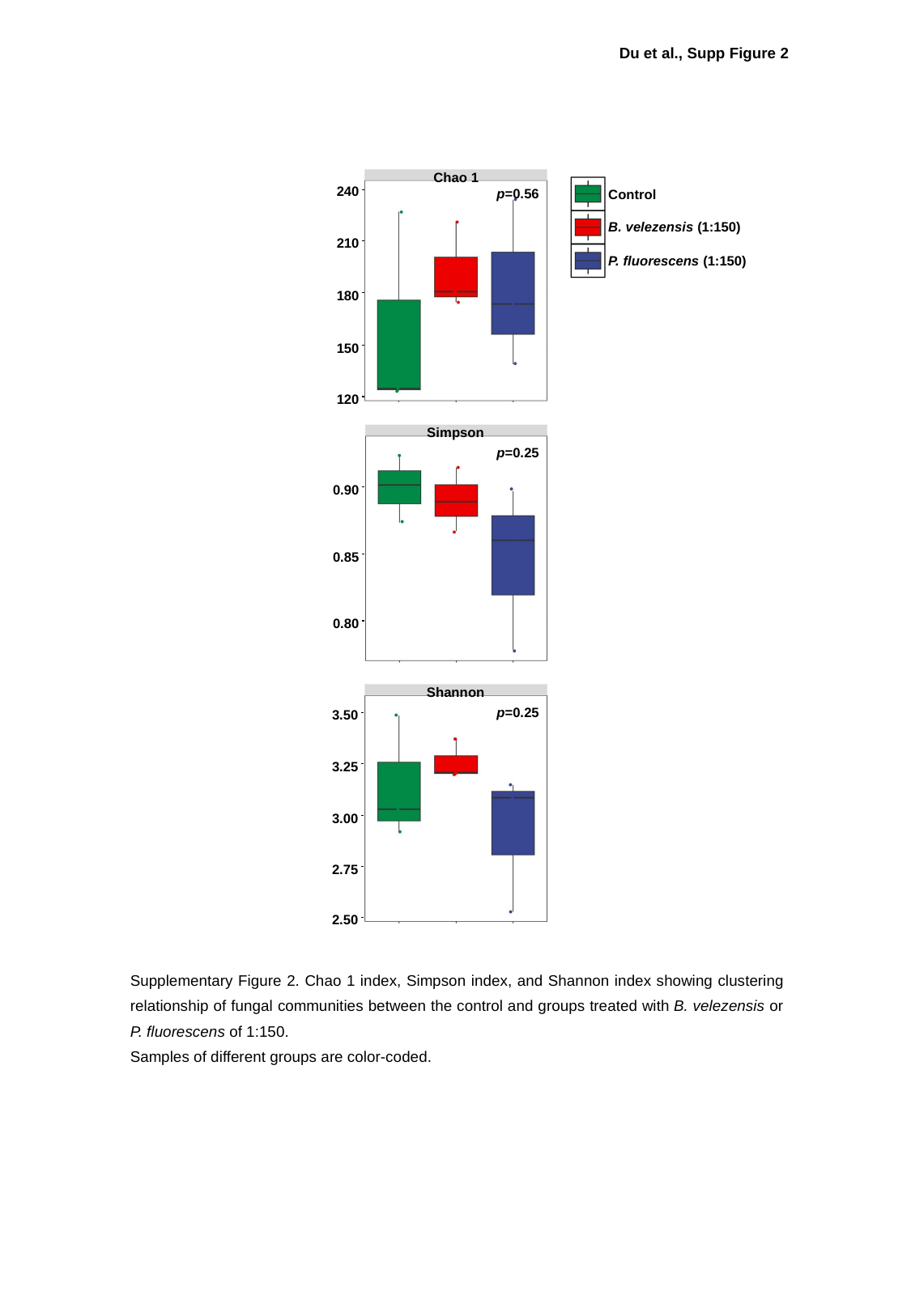

Du et al., Supp Figure 2
Chao 1
240
p=0.56
Control
B. velezensis (1:150)
210
P. fluorescens (1:150)
180
150
120
Simpson
p=0.25
0.90
0.85
0.80
Shannon
p=0.25
3.50
3.25
3.00
2.75
2.50
Supplementary Figure 2. Chao 1 index, Simpson index, and Shannon index showing clustering relationship of fungal communities between the control and groups treated with B. velezensis or P. fluorescens of 1:150.
Samples of different groups are color-coded.

## Slide 3
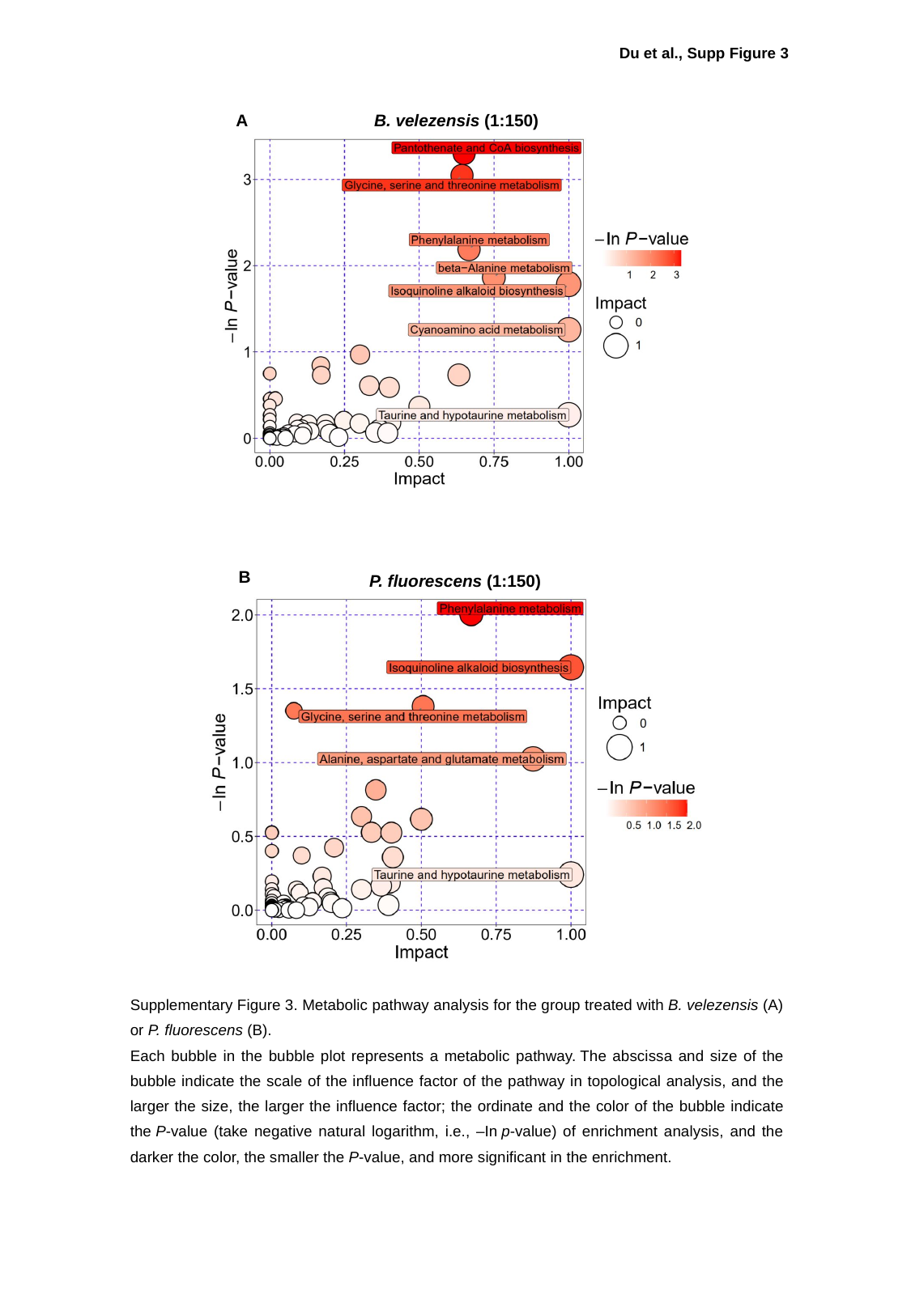

Du et al., Supp Figure 3
A
B. velezensis (1:150)
B
P. fluorescens (1:150)
Supplementary Figure 3. Metabolic pathway analysis for the group treated with B. velezensis (A) or P. fluorescens (B).
Each bubble in the bubble plot represents a metabolic pathway. The abscissa and size of the bubble indicate the scale of the influence factor of the pathway in topological analysis, and the larger the size, the larger the influence factor; the ordinate and the color of the bubble indicate the P-value (take negative natural logarithm, i.e., –In p-value) of enrichment analysis, and the darker the color, the smaller the P-value, and more significant in the enrichment.
